# Association of MCP-1 -2518 A/G Single Nucleotide Polymorphism with the Serum Level of CRP in Slovak Patients with Ischemic Heart Disease, Angina Pectoris, and Hypertension

**DOI:** 10.1155/2009/390951

**Published:** 2009-07-22

**Authors:** Maria Bucova, Jan Lietava, Peter Penz, Frantisek Mrazek, Jana Petrkova, Marian Bernadic, Martin Petrek

**Affiliations:** ^1^Institute of Immunology, Faculty of Medicine, Comenius University, 811 08 Bratislava, Slovakia; ^2^Department of Internal Medicine II, Faculty of Medicine, Comenius University, 813 69 Bratislava, Slovakia; ^3^Department of Internal Medicine I, Faculty of Medicine, Comenius University, 813 69 Bratislava, Slovakia; ^4^Laboratory of Immunogenomics, Department of Immunology, Faculty of Medicine, Palacky University, 77 900 Olomouc, Czech Republic; ^5^Department of Internal Medicine I, Faculty Hospital, 77 900 Olomouc, Czech Republic; ^6^Institute of Pathological Physiology, Faculty of Medicine, Comenius University, 811 08 Bratislava, Slovakia

## Abstract

The aim of our work was to find if MCP-1 -2518 (A/G) single nucleotide polymorphism (SNP) influences somehow the serum concentrations of high-sensitive CRP (hsCRP) both in patients suffering from ischemic heart disease (IHD), myocardial infarction (MI), angina pectoris (AP), and hypertension (HT) and in control group of healthy subjects. Totally, 263 patients with the diagnosis of IHD, out of them 89 with MI, 145 with AP, 205 with HT, and also 67 healthy subjects were included in the study. First, we estimated the serum levels of hsCRP. We found that patients with AP had significantly higher serum level of hsCRP than both control group of healthy subjects (*P* = .043) and IHD patients without AP (*P* = .026). The presence of the mutant G allele statistically significantly correlated with the higher serum levels of hsCRP in patients with IHD (*P* = .016), AP (*P* = .004), and HT (*P* = .013). Higher correlations were found in men (AP: *P* = .019; HT: *P* = .047). In all cases the highest levels of hsCRP were found both in patients and healthy controls with homozygous GG genotype.

## 1. Introduction

Inflammation is a bodily response to tissue injury or irritation and primarily facilitates restoration of tissue health. It can be induced not only by infection but also by processes associated with any type of cell, tissue, or organ damage.

Chronic low-grade inflammation has an important role also in the ethiology of coronary heart disease (CHD) [1–3]. Several studies have shown that elevated plasma levels of C-reactive protein (CRP), interleukin-6 (IL-6), fibrinogen and other soluble inflammatory mediators are associated with both the severity of atherosclerosis and the risk of CHD [4–7].

There is a possibility that individuals vary in their sensitivity to the general background of intercurrent low-grade acute-phase stimuli to which everybody is exposed, and that those who are higher “CRP responders” through genetic and/or acquired mechanisms are also more susceptible to progression and complications of atherosclerosis [8].

The aim of our work was to find if MCP-1 -2518 (A/G) single nucleotide polymorphism (SNP) influences somehow the tested concentrations of hsCRP both in patients suffering from ischemic heart disease (IHD), myocardial infarction (MI), angina pectoris (AP), and hypertension (HT) within the cohort of IHD patients and in control group of healthy subjects. The reason for our intention was that the chemokine MCP-1/CCL2 belongs to key inflammatory CC chemokines playing central role in atherosclerosis and cardiovascular disease development—disease states guided with chronic low-grade inflammation [9–11]. MCP-1/CCL2 is a potent chemoattractant for monocytes, T cells, and NK cells. MCP-1 induces the transmigration of CCR2^+^ monocytes from the circulation, promotes their differentiation to lipid laden macrophages, and contributes to the proliferation of arterial smooth muscle cells. This chemokine plays a dual role in myocardial ischaemia. In addition to several negative roles in the process of atherosclerosis, thrombotic occlusion of coronary artery, and in the process of reperfusion, this chemokine protects myocytes from hypoxia-induced cell death and has also positive effect in myocardial infarct healing [12, 13].

## 2. Materials and Methods

### 2.1. Patients and Controls

Our case control study with determined MCP-1 polymorphism comprised 263 patients with IHD (118 males/145 females, mean age 61.52 ± 10.173/64.16 ± 7.543; *P* = .02), out of them 89 with MI (55 males/34 females, mean age 59.71 ± 10.031/64.62 ± 6.448 years; *P* = .006) as well as 145 with angina pectoris (AP) (59 males/86 females, mean age 60.71 ± 9.837/64.09 ± 7.575 years, *P* = .028). The control group comprised of 67 healthy subjects from a region Velky Lom in middle Slovakia (36 males/31 females, mean age 50.33 ± 10.690 years/49.71 ± 9.353 years. *P* = .802). They have the same MCP-1 gene prevalence as healthy subjects from whole Slovakia tested before [10, 14]**.** Patients and control subjects were unrelated and of Caucasian origin. The study was approved by the local Ethics Committee (Faculty of Medicine Comenius University, Bratislava), and all subjects signed an informed consent.

The original cohort of patients was enrolled in 1999-2000 for the Homocystein Study. Patients with ischemic heart disease were recruited from cardiological register of Cardiological laboratory of the second Department of Internal Medicine, Comenius University in Bratislava (standardized mortality 9.8), cardiological registers of two specialists in Nove Zamky (stardardized mortality 10.4), and one register in Velky Lom (standardized mortality 12.6). Randomization was made according to random tables by independent researchers. Basic characteristics (structure of sex, age, and diseases) were analyzed in both groups (patients recruited into project and patients excluded). Subjects in whom ischemic heart disease (according to below mentioned criteria) was diagnosed were considered as patients; apparently healthy subjects were considered as controls.

Ischemic heart disease (IHD) was defined as documented myocardial infarction (hospitalization or coronarography), or presence of documented typical angina pectoris (ECG or Holter) or documented silent ischemia (ECG, Holter, ECG or exercise test), or pathological finding on coronary arteries during selective coronarography or other interventions. Typical angina pectoris was defined as substernal chest discomfort with a characteristic quality and duration that is provoked by exertion or emotional stress and is relieved by rest or nitroglycerin [15]. It was also documented by ECG ST segment deviation (exercise test, Holter, or ECG at rest) reacting to antianginal therapy. Arterial hypertension was defined as increased blood pressure ≥140/90 mm Hg measured using standard protocol or normotension on antihypertensive therapy. Blood pressure was measured in a separate quiet room after 5 minutes in sitting position three times by sphygnomanometer using auscultatory method.

Cardiovascular symptomatology in control subjects was evaluated according to the standard Rose questionnaire [16] and, additionally, in 55% of control subjects by exercise electrocardiography.

Complete personal and medical history was taken by qualified physicians, who underwent training to obtain standardized data. Analyzed parameters included whole blood count, complete lipid profile (total cholesterol, triglycerides, apoprotein B, apoprotein AI, high-density lipoprotein-cholesterol, low-density lipoprotein-cholesterol), glucose, total antioxidant status, homocysteine and vitamins B status, inflammatory markers, and several oxidative stress parameters.

### 2.2. Assessment of MCP-1 -2518 SNP

Genomic DNA was extracted using a standard salting out procedure [17]. MCP-1 wild-type (A) and mutant (G) alleles were typed by polymerase chain reaction using sequence specific primers (PCR-SSP). Two reaction formats with specific reactions either to A or G allele of the MCP-1 **-**2518 SNP were used, and an internal control was adopted from phototyping [14]. The sequences of specific primers were allele A, forward: 5′GTG GGA GGC AGA CAG CTA; allele G, forward: 5′ GTG GGA GGC AGA CAG CTG; constant reverse: 5′TGA GTG TTC ACA TAG GCT TC. The PCR mixture was according to Phototyping [18]. PCR amplification was carried out using a PTC-100 Thermal Cycler (MJ Research, Inc., Waltham, Mass, USA). The cycling protocol was described earlier [19]. MCP-1 -2518 genotypes were assessed from the presence/absence of PCR amplicons specific to the particular alleles in a standard 2% agarose gel stained with ethidium-bromide.

### 2.3. CRP Level Determination

The serum level of CRP in patients and control subjects was determined turbidimetrically by an ADVIA instrument (Bayer Corporation, Tarrytown, New York, USA) with reagents from Randox Laboratories, Ltd. (Crumlin, UK).

### 2.4. Statistics

The populations were tested for conformity to the Hardy-Weinberg equilibrium using a 2 × 2 *χ* ^2^ test between observed and expected numbers. Levels of hsCRP between controls and (sub)groups of patients were compared by Mann-Whitney U-test. The association of genotypes with the levels of hsCRP was statistically evaluated by Kruskal-Wallis test, and for comparison of hsCRP concentrations to particular genotypes the Bonferonni Post Hoc test was used. A *P*-value < .05 was considered to be significant.

## 3. Results

### 3.1. Serum Levels of hsCRP in Patients with IHD, MI, AP, and HT, and in Control Group of Healthy Subjects

The serum levels of hsCRP in patients with estimated diagnosis were higher than those in control group of healthy subjects. The highest levels of hsCRP were found in patients with AP (*P* = .043). These levels were statistically significantly higher than those in IHD patients without AP (*P* = .026) (Table 1).

**Hardy-Weinberg Equilibrium**

The healthy control group was in Hardy-Weinberg equilibrium (HWE) with regard to the distribution of the MCP-1 -2518 A/G genotypes (*P* > .05); observed MCP-1 -2518 G allele frequency was similar to the data reported previously from other Caucasian populations [20]. However, IHD patients were slightly deviated from HWE due to the higher frequency of GG genotype and lower frequency of AG heterozygotes (*P* = .02). Similarly, subgroups of IHD patients with MI (*P* = .04) and AP (*P* = .00002) were not in HWE.

### 3.2. Concentrations of hsCRP in the Sera of Patients with IHD, MI, AP, and HT in Relation to MCP-1 -2851 (A/G) SNP Polymorphism and to Sex

The tested SNP of MCP-1 gene, concretely the presence of the mutant G allele, statistically significantly correlated with the higher serum level of hsCRP in patients with IHD (*P* = .016; in men: *P* = .060; in women: *P* = .197), AP (*P* = .004; in men: *P* = .019; in women: *P* = .091) and HT (*P* = .013; in men: *P* = .047; in women: *P* = .122, Tables 2  and 3).

The association between the serum level of hsCRP with the tested MCP-1 -2518 (A/G) SNP was not statistically significant either in patients with MI (*P* = .494) or in healthy control subjects (*P* = .787).

**Bonferroni's Post hoc Test (Correlations between Individual Genotypes with the Level of hsCRP in Serum of Patients and Healthy Subjects)**

This test showed differences in the serum levels of hsCRP comparing subjects with typical genotypes—AA : AG, AG : GG, and AA : GG. We found statistically significant differences in patients with IHD (AG : GG; *P* = .022, AA : GG; *P* = .018), AP (AG : GG; *P* = .003, AA : GG; *P* = .012), and HT (AG : GG; *P* = .016, AA : GG; *P* = .016) (Table 2).

**Bonferroni's Post hoc Test (Correlations between Individual Genotypes with the Level of hsCRP in Serum of Patients and Healthy Subjects According to Sex)**

We revealed high significant correlations in men with AP (AG : GG; *P* = .037, AA : GG; *P* = .026) and in men with HT (AA : GG; *P* = .044) (Table 3).

## 4. Discussion

Understanding the factors that directly or indirectly regulate the CRP release at baseline and during inflammation is very important in context of coronary risk prediction. More scientific groups studied CRP gene polymorphisms and found that basal levels of CRP both in patients and healthy controls are genetically determined and under repeated examination in healthy subjects relatively stable. Thus understanding the genetic background of CRP that regulates basal but also by infection or any type of inflammation-induced concentration of CRP might contribute to stratification of healthy subjects to different groups with higher or lower degree of cardiovascular disease development [21–24]. D'Aiuto et al. [25] found that patients with homozygous +1444TT allele of CRP gene had significantly higher serum level of CRP induced by inflammatory stimulus. The production of CRP is, except of CRP gene, regulated also by other genes coding for IL-6, IL-1 beta, and IL-1Ra [26, 27].

The human CRP gene lies on chromosome 1, within a conserved region that encodes for proteins critical to the immune system and to intercellular communication [6, 28]. Dupuis et al. [29] found that multiple genes on chromosome 1 may influence inflammatory biomarker levels and may have a potential role in development of cardiovascular disease. They hypothesized that production of biomarkers of vascular inflammation is modulated genetically and by environmental factors.

In the first part of our study we compared the serum levels of hsCRP in patients with IHD, AP, MI, and HT with the level in control subjects and found that serum levels of hsCRP in patients with AP were higher than those in control group of healthy subjects (*P* = .043). These levels were higher also in comparison to IHD patients without AP (*P* = .026) (Table 1).

The tested MCP-1 -2518 (A/G) SNP, concretely the presence of the mutant G allele, statistically significantly correlated with the higher serum level of hsCRP in patients with IHD (*P* = .016), AP (*P* = .004), and HT (*P* = .013), and in all cases higher correlations were found in men (Table 3). Comparing subjects with typical genotypes (AA, AG, GG), Bonferroni's post hoc test showed differences in the serum levels of hsCRP in patients with IHD, AP, and HT. The highest levels of hsCRP were associated with the presence of homozygous GG genotype (Table 2). The test also revealed high significant correlations of the presence of G allele with the elevated levels of hsCRP in men with AP and HT.

## 5. Conclusion

Our results suggest that MCP-1 -2518 (A/G) SNP is associated with the level of hsCRP in patients with ischemic heart disease, angina pectoris, and hypertension in the Slovak population. The highest levels of hsCRP were found in subjects with homozygous GG genotype.

## Figures and Tables

**Table 1:** Serum levels of hsCRP in patients with IHD, MI, AP, and hypertension and in control group of healthy subjects. Legend: IHD: ischemic heart disease; MI: myocardial infarction; AP: angina pectoris; HT: hypertension.

Estimated groups	Number of subjects	hsCRP (ng·ml^−1^) ± SD	Significance (Mann-Whitney) (*P* = )
Controls without HT	62	2.149 ± 2.348	
IHD	299	2.607 ± 2.663	.199
MI	102	2.671 ± 2.891	.228
AP	159	2.899 ± 2.794	.043
HT (in IHD patients)	229	2.541 ± 2.479	.214
Angina pectoris versus IHD^+^AP^− ^	159/140	2.899 ± 2.794	.026
		2.275 + 2.474	

**Table 2:** Serum levels of hsCRP in patients with IHD, MI, AP, and HT in relation to the tested MCP-1 -2851 (A/G) SNP. Legend: IHD: ischemic heart disease; MI: myocardial infarction; AP: angina pectoris; HT: hypertension; Sg.: significance; SD: standard deviation.

Estimated groups	Number of subjects	Genotype	hsCRP (ng·ml^−1^) ± SD (men + women)	Compared genotypes	Bonferroni's post hoc test (*P* = )
Controls	37	AA	2.323 ± 2.4126	AA : AG	1.000
	25	AG	2.650 ± 1.9339	AG : GG	1.000
	5	GG	2.992 ± 4.7494	AA : GG	1.000
		Sg.:	*P* = .787		

IHD	142	AA	2.449 ± 2.6945	AA : AG	1.000
	93	AG	2.423 ± 2.2847	AG : GG	.022
	28	GG	4.004 ± 3.8814	AA : GG	.018
		Sg.:	*P* = .016		

MI	50	AA	2.573 ± 2.7242	AA : AG	1.000
	29	AG	2.503 ± 2.5237	AG : GG	.786
	10	GG	3.682 ± 4.1514	AA : GG	.792
		Sg.:	*P* = .494		

AP	83	AA	2.560 ± 2.6080	AA : AG	1.000
	40	AG	2.615 ± 2.5862	AG : GG	.003
	22	GG	4.833 ± 3.9930	AA : GG	.012
		Sg.:	*P* = .004		

HT	111	AA	2.375 ± 2.5071	AA : AG	1.000
	72	AG	2.308 ± 2.1775	AG : GG	.016
	22	GG	4.054 ± 3.6638	AA : GG	.016
		Sg.:	*P* = .013		

**Table 3:** Serum levels of hsCRP in patients with IHD, MI, AP, and HT in relation to the tested MCP-1 -2851 (A/G) SNP and to sex. Legend: IHD: ischemic heart disease; MI: myocardial infarction; AP: angina pectoris; HT: hypertension; Sg.: significance; SD: standard deviation.

Estimated groups	Genotype	Serum levels of hsCRP		Compared genotypes	Bonferroni's	
	men/women	(ng·ml^−1^) ± SD			post hoc test	
		Men	Women		Men	Women
					(*P* = )	(*P* = )

Controls	AA (15/22)	1.873 ± 2.0712	2.630 ± 2.6220	AA : AG	1.000	
	AG (17/8)	2.085 ± 1.5630	3.850 ± 2.1950	AG : GG	.760	
	GG (4/1)	3.603 ± 5.2527	0.550 ± .	AA : GG	.598	
	Sg.:	*P* = .426	*P* = .338			

IHD	AA (61/81)	2.402 ± 2.3681	2.484 ± 2.9305	AA : AG	1.000	1.000
	AG (43/50)	2.230 ± 2.0841	2.589 ± 2.9305	AG : GG	.065	.334
	GG (14/14)	4.016 ± 3.8739	3.993 ± 4.0351	AA : GG	.093	.224
	Sg.:	*P* = .060	*P* = .197			

MI	AA (29/21)	2.532 ± 2.4858	2.629 ± 3.0863	AA : AG	1.000	1.000
	AG (18/11)	1.793 ± 1.4071	3.665 ± 3.4750	AG : GG	.143	1.000
	GG (8/2)	4.016 ± 4.4663	2.345 ± 3.3163	AA : GG	.467	1.000
	Sg.:	*P* = .138	*P* = .668			

AP	AA (34/49)	2.508 ± 2.2623	2.597 ± 2.8456	AA : AG	1.000	1.000
	AG (14/26)	2.002 ± 1.5980	2.944 ± 2.9636	AG : GG	.037	.088
	GG (11/11)	4.799 ± 4.0405	4.866 ± 4.1418	AA : GG	.026	.255
	Sg.:	*P* = .019	*P* = .091			

HT	AA (41/70)	2.209 ± 1.9115	2.472 ± 2.8065	AA : AG	1.000	1.000
	AG (27/45)	1.918 ± 1.5853	2.541 ± 2.4529	AG : GG	.090	.133
	GG (9/13)	3.820 ± 3.1319	4.216 ± 4.1089	AA : GG	.044	.191
	Sg.:	*P* = .047	*P* = .122			
